# A Rare Complication of Noncompliance Status Post-Transhiatal Esophagectomy and Esophago-Gastroanastomosis

**DOI:** 10.1155/2020/8833110

**Published:** 2020-11-16

**Authors:** Daniel Ramirez, Graham Appelbe, Venkatramana Vattipally, Justin Miller

**Affiliations:** ^1^Department of Internal Medicine, Ascension Genesys Medical Center, Grand Blanc, MI, USA; ^2^Department of Radiology, Ascension Genesys Medical Center, Grand Blanc, MI, USA; ^3^Department of Gastroenterology, Ascension Genesys Medical Center, Grand Blanc, MI, USA

## Abstract

Gastropleural fistulas are a complication of peptic ulcers in hiatal hernias, trauma, infections, surgical complications, and malignancy. Presenting symptoms may include gastric and chest pain with respiratory failure in the setting of pneumonitis, hydropneumothorax, or tension pneumothorax. We describe a 57-year-old male with a history of transhiatal esophagectomy and esophago-gastroanastomosis who presented in the setting of dyspnea and dark orogastric tube output. Upper endoscopy revealed multiple gastric ulcers with a dominant ulceration communicating with an adjacent space, and a fistulous tract was demonstrated on computed tomography chest, confirming a gastropleural fistula, a rare life-threatening condition.

## 1. Introduction

Gastropleural fistulas (GPFs) are a rare and dangerous complication of different pathologies including peptic ulcer disease, trauma, infections, surgical complications, and malignancy [1]. Presentation is typically insidious, and symptoms may include abdominal and chest pain, respiratory failure, and signs of septic shock [1–7]. Treating physicians should have a high index of suspicion with high-risk patients, as prompt diagnosis is essential to decrease mortality in this life-threatening diagnosis. We describe a 57-year-old male with a history of transhiatal esophagectomy and esophago-gastroanastomosis secondary to high-grade dysplasia who presented in the setting of dyspnea and dark orogastric tube (OGT) suction. Respiratory distress worsened, and the patient was emergently intubated in the Emergency Room. Bronchoscopy revealed clear airways; however, esophagogastroduodenoscopy (EGD) revealed multiple gastric ulcers with a dominant ulceration communicating with an adjacent space. Computed tomography (CT) of the chest confirmed suspicion of fistulous tract, and this patient was diagnosed with the rare condition of GPF requiring urgent correction with endoscopic suture placement.

## 2. Case Report

The patient was a 57-year-old male with a history of COPD and transhiatal esophagectomy with esophago-gastroanastomosis and J-tube placement (2005) secondary to high-grade dysplasia within a segment of Barrett's esophagus. Since 2005, he had numerous hospitalizations for dysphagia, regurgitation, and aspiration with subsequent balloon dilation without significant relief. Several records indicate a significant history of leaving against medical advice (AMA) and noncompliance with tobacco abuse and eating solid foods leading to multiple EGD with subsequent removal of retained food products. Also noted on previous endoscopies were findings suggesting functional gastric outlet obstruction and multiple Forrest class III gastric ulcers thought to be from ischemia and stasis.

Prior to this admission, the patient ingested solid food which induced abdominal pain, nausea and nonbloody, nonbilious emesis. In the ER, the patient became dyspneic requiring intubation for airway protection. OGT suctioned approximately 1200 cc of dark-colored fluid. Pertinent physical findings included cachectic appearance with right-sided coarse lung sounds. The abdomen was nondistended, soft, and nontender, and without organomegaly or masses. The J-tube was in place without discharge or erythema.

Chest X-ray showed right middle and lower lobe infiltrate. Labs were significant for leukocytosis (WBC 22.9 K/cmm), anemia (hemoglobin of 8.8 g/dL), thrombocytosis (platelets greater than 1000 K/cmm), and lactic acid of 9.92 mmol/L. Electrolyte abnormalities included sodium of 129 mmol/L, chloride of 75 mmol/L, and hyperglycemia with glucose at 248 mg/dL. Arterial blood gas showed hypercapnia without significant hypoxia, and EKG showed sinus tachycardia without ischemic changes. He received a 30 mL/kg bolus of intravenous fluids, antibiotics including vancomycin/cefepime with the addition of clindamycin for aspiration coverage, and 80 mg IV pantoprazole and was admitted to the Intensive Care Unit (ICU). Due to worsening hypoxia and the suspicion of aspiration, Critical Care performed bronchoscopy which showed a normal-appearing trachea as well as clear bilateral lung lobes. Secretions were suctioned and the right lung wash-out specimens were sent for pathology; however, there were no reported findings of food material or bleeding. Gastroenterology was consulted for concern of upper GI bleed with dark liquid from OGT and a decrease in Hgb from 8.8 to 6.2 g/dL requiring 2 units of packed red blood cells (pRBCs).

**Figure 1:**
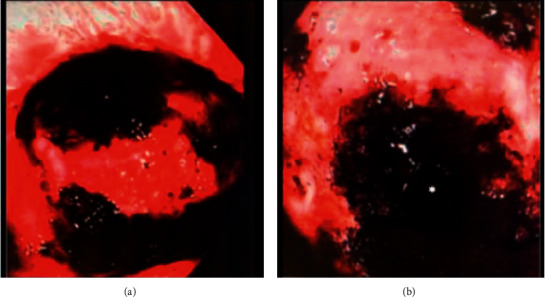
(a) Dominant ulceration along the greater curvature. (b) Image within ulceration revealing fistula opening leading to pleural space marked by the asterisk.

**Figure 2:**
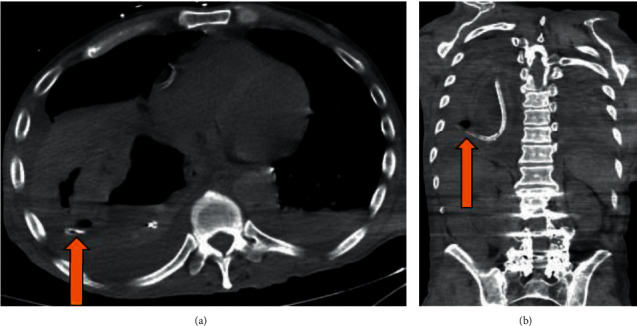
The arrow in (a) and (b) demonstrate a catheter passing from the stomach into the pleural cavity consistent with gastropleural fistula.

EGD discovered 1.5 L of dark gastric content that was suctioned revealing 5 clean-based gastric ulcers. One such ulcer measured approximately 2 × 2.5 cm in diameter and approximately 1 cm deep at 44 cm from the incisors, along the greater curvature. At the base of this ulcer appeared an external communication with a questionable fistula (Figure 1). Biopsies obtained revealed inflammatory granulation tissue without signs of malignancy. Subsequent insertion of the gastric tube after endoscopy was completed, and follow-up CT imaging (Figure 2) showed right-sided gastric pull-through with a concerning fistulous tract connecting to the underlying lung parenchyma, as the distal tip of the gastric feeding tube terminated within a focal air lucency within the lung parenchyma. A large consolidation with right-sided pleural effusion was also identified, likely contributing to this patient's worsening respiratory status. Due to these concerning findings, a decision was made to transfer this patient to a tertiary center where he was known to the cardiothoracic surgical group for further surgical management. The patient was subsequently treated with endoscopic suturing and continued on antibiotic therapy; however, despite treatments, he succumbed to his illness several months later.

## 3. Discussion

First described in 1960 by Markowtts and Herter, GPF can be an acquired complication from peptic ulcerations via hiatal hernia, trauma, and perforation with abscess formation [1]. Case reports have described oral intake of steroids or anti-inflammatory drugs increasing the risk of gastric perforations, and several studies have demonstrated that GPF can also be a serious complication from surgical interventions and malignancy [2–6]. As the diaphragm acts as an effective barrier, communicating fistulas from the stomach and pleural space through the diaphragm are extremely rare. Diagnosis is normally via contrast radiology, upper endoscopy, and/or via open operation.

Prognosis of this rare complication depends on the time of diagnosis and intervention with prolonged use of antibiotics, acid-suppressing medications, and strict NPO with total parenteral nutrition which can result in successful outcomes. In the absence of universal guidelines, there are conflicting reports on the proper care of GPF. A majority of reports agree that the laparoscopic approach and surgical interventions are definitive treatments as data indicate higher mortality with conservative management [7–9]. Meta-analysis of several case reports and retrospective studies has evaluated the outcomes of various surgical and endoscopic interventions. Stent placement, endoscopic clipping techniques, or surgical sealants have all shown high success rates of repair with decreased morbidity and mortality when compared to nonsurgical therapies [8, 9]. Contemporary studies have shown improving trends with fistula repairs with evidence implying that complex endoscopic surgical treatments or combined treatment with simultaneous or sequential endoscopic methods can optimize management [8]. While postoperative complication management is crucial, preoperative modifiable factors have also been investigated. Several reviews have implicated potential quality improvements to postsurgical patients and distinguished tobacco abuse and hyperobesity as modifiable risk factors and that as the quantity and chronicity of tobacco use rose, it showed positive correlation to postsurgical complications [10–12].

This case provides an excellent example of septic shock in the setting of an extremely rare surgical and traumatic complication. GPF are infrequent and life-threatening with insidious onset and nonspecific symptoms. This diagnosis has been historically difficult to establish and is often misdiagnosed, as is noted in several reviews and case reports [2, 5–7]. A high index of suspicion should be held in patients with significant risk factors including previous surgeries, malignancy, and gastric ulcerations. Providing awareness of this uncommon pathology may help eliminate delay of diagnosis, as rapid surgical or endoscopic correction appears to be the definitive form of treatment. In the case of our patient, the combination of esophago-gastroanastomosis with gastric pull-through and the chronic history of tobacco abuse and NPO noncompliance likely led to persistent inflammation at the numerous gastric ulcer sites eventually leading to this devastating complication.
